# Social Media Use, Affect, and Dietary Choices Across Age Groups—Insights from the Special Issue “The Impact of Social Media on Eating Behavior”

**DOI:** 10.3390/nu18020335

**Published:** 2026-01-21

**Authors:** María del Mar Uclés-Torrents, Gema Esperanza Ruiz-Gamarra, Pilar Aparicio-Martínez

**Affiliations:** 1 Nursing, Pharmacology and Physiotherapy Department, University of Cordoba, 14004 Cordoba, Spain; 2 GE10 Clinical and Epidemiological Research in Primary Care (GICEAP), Maimonides Biomedical Research Institute of Cordoba (IMIBIC), 14004 Cordoba, Spain; 3 Palma del Río Health Center, Dr. Trujillo Street, Palma del Río, 14700 Cordoba, Spain

## 1. Introduction

Social connection is a key pillar of human life that is instrumental to the health and well-being of all, regardless of culture, ethnicity, or age. Social media (SM), also known as social networking sites, are digital platforms where users build communities to share information, ideas, personal messages, images, and videos. Platforms such as Facebook, X (formerly Twitter), Instagram, and TikTok were designed with distinct functionality and affordances, and are now widely used for content creation, distribution, and engagement, with TikTok being the most popular in recent years [1]. These platforms, first developed in the early 20th century, are used by multiple users, including professionals, elderly adults, and even underage users. Social media use (SMU) has been examined across diverse domains, including emotion and psychological functioning [2]. Recent work clarifies that SMU is not monolithic: common typologies distinguish belief-based use (for example, sharing opinions), comparison-based use (for instance, body comparison), image-based use (such as monitoring likes), and consumption-based use (like watching videos), and these types are differentially associated with affective outcomes [3,4].

## 2. Eating Behavior and Social Media Exposure

Across children, adolescents, young adults, and even adults, social media exposure is consistently associated with unfavorable dietary patterns. These include a higher consumption of unhealthy snacks, sugar-sweetened beverages, and fast food; a lower intake of fruits and vegetables; and an increased likelihood of meal skipping [5,6]. Notably, multiple reviews report that these associations are broadly consistent across age groups once access to social media is established, with effects frequently described as being independent of age [6]. While deleterious outcomes predominate, positive effects are documented when exposure involves peer-generated healthy eating content rather than influencer content, or when social media is deliberately used in structured health promotion interventions [7,8,9,10,11].

According to a 2019 randomized controlled trial involving 176 children aged 9–11 years, those exposed to social media influencers promoting unhealthy snacks consumed significantly more total calories, as well as more calories from unhealthy snacks, compared with children exposed to non-food content. In children aged 10–16 years, watching food brand videos on YouTube increased unhealthy food scores by 0.46 points (*p* = 0.015), drink scores by 0.34 points (*p* = 0.009), and combined scores by 0.80 points (*p* = 0.003); seeing favorite food brands advertised online increased food scores by 0.63 points (*p* = 0.011), and purchasing food online increased combined scores by 0.71 points (*p* = 0.009) [6]. In contrast to the aforementioned findings in Western populations, the study by Januraga and colleagues focused on the link between body image, nutritional adequacy, and fad diets among female adolescent models. Although conducted in Indonesia, the study was limited to the modeling community and did not specifically examine the impact of social media exposure on food choices and nutrient intake across the general Indonesian population, but did indicate that a campaign based on SM created awareness among young females related to healthy and unhealthy habits [12]. Exposure to core (healthy) food messages has been shown to increase vegetable consumption via food literacy as a mediating mechanism (indirect effect: 0.01, SE 0.003, *p* < 0.001) [10]. Among adolescents, exposure to peers’ videos on healthy eating increased vegetable intake (*p* < 0.05), whereas influencer content did not produce comparable effects, suggesting that social proximity moderates the impact [10,13]. Moreover, social media interventions have yielded statistically significant improvements in diet quality, including increases in fruit and vegetable intake and Healthy Eating Index scores; these gains are derived from structured interventions rather than passive exposure [13,14].

Beyond specific food intake, social media engagement has been linked to problematic eating patterns. Associations with skipping breakfast are repeatedly reported, and there is evidence tying social media usage to disordered eating behaviors, including restrictive eating, binge eating, and orthorexia nervosa, often connected to body image dissatisfaction [5,13]. Social media engagement is also associated with body dissatisfaction, dieting/restricting food, overeating, and, paradoxically, choosing healthy foods; the direction of effect depends on the nature of engagement. Negative engagement behaviors, such as reassurance seeking or maladaptive patterns, correlate with higher body dissatisfaction and disordered eating [5].

The existing literature provides limited insight into age-specific differences in these effects across the lifespan, ranging from childhood to older adulthood (6–50 range age). Multiple systematic reviews explicitly report that associations between social media exposure and unhealthy dietary patterns are independent of age within the 2–18 year range [6]. According to recent research, most studies have focused on narrow age groups or have statistically adjusted for age rather than analyzing age as a moderating factor. Notably, a large study found that 69.5% of 11-to-15-year-olds had at least one social media account, and a majority of those under age 13 also reported using social media, while 70.6% of older children have social media accounts, specifically Facebook or other similar platforms, compared with 12.3% of younger children [7]. Nuanced developmental influences are evident in source dynamics: peers tend to influence adolescents’ vegetable intake, whereas parents influence younger children’s nutrition knowledge; young adults (18–30 years) have reported higher negative body image scores associated with Facebook exposure than older cohorts [5].

Physiologically, exposure to unhealthy (versus healthy) digital food images heightens activity in reward and attention networks among children and adolescents; these responses are moderated by appetitive state (hunger vs. satiation) and by the portion size and energy density of the depicted foods [6]. Food marketing can interfere with the neural processing of food cues, biasing attention and choice toward high-reward items [6]. Social mechanisms are prominent: descriptive norms mediate the relationship between exposure to non-core (unhealthy) food content and non-core intake, and advertising via influencers and peers constitutes a key social pathway [10,13,15]. According to an analysis by Kulandaivelu and colleagues, Instagram content offers several opportunities to support and enhance food literacy education, thereby improving healthy eating behaviors in reading, meal planning, and cooking [10]. Psychological mechanisms involve body image dissatisfaction and social comparison processes; body image is significantly associated with food selection, confounding the exposure–diet relationship [9]. Additionally, many users seek external validation and modify their appearance to conform to perceived ideals despite recognizing potential harms [5].

The apparent contradiction between studies reporting negative dietary effects and those demonstrating positive outcomes can be reconciled primarily through exposure type and mechanism. Passive exposure to food marketing, brand content, and unhealthy food images is consistently associated with the increased consumption of unhealthy foods across all age groups, operating via perceived descriptive norms that normalize non-core foods among peers [6,7,9]. Conversely, structured health promotion interventions and peer-generated healthy content exert beneficial effects by enhancing food literacy, enabling more well-informed dietary decisions [9,10,11]. These two mechanistic pathways, non-core exposure via descriptive norms and core exposure via food literacy, likely operate regardless of age, providing a coherent explanation for the recurring observation that effects are independent of age. The scarcity of significant age differences may reflect both shared mechanisms across developmental stages once social media use begins and methodological constraints that obscure true developmental variations, including narrow age ranges and the frequent statistical control of age rather than stratified analyses [3,6]. Nonetheless, exposure intensity differs markedly by age, implying that even if per-exposure effects do not vary, long-run impacts may diverge due to cumulative exposure [7].

Effect estimates should be interpreted in light of the studied population and context. Studies conducted in Indonesia, Belgium, Australia, and various Western nations may reflect culturally specific food environments and platform norms [6,9,10]. The predominance of cross-sectional designs constrains causal inference, and the reliance on self-reported dietary measures introduces risks of recall and social desirability bias [6,9,10,11,12,14]. The intervention literature indicates that social media can serve as an effective platform for dietary behavior change when deliberately designed with techniques such as social support, goal setting, self-monitoring, and feedback; success appears to depend less on age per se than on appropriate platform selection and intervention design aligned with the target population’s usage patterns [11,14].

## 3. Insight into This Special Issue

Despite these findings, significant research gaps remain regarding the impact of social media on eating behavior across diverse age groups. This Special Issue addresses these gaps by presenting new evidence from various age groups and regions. Five papers are included in this issue:

The observational study, conducted in Spain, by Pérez-Jiménez et al. (2025), examined the relationship between social media use, content exposure, and the risk of developing disordered eating behaviors among adolescents. The study found that higher levels of social media consumption, particularly engagement with appearance- and weight-focused content, were significantly associated with increased eating disorder symptomatology in middle- and high-income adolescents. The participants who displayed signs of social media addiction were at especially high risk, underscoring the role of compulsive social media use as a key element in adolescent disordered eating (Contribution 1).

In contrast, the study by Chen et al. (2025), carried out in Taiwan, highlighted the potential benefits of social media when content is educational and skill-based. Their study investigated the influence of Instagram cooking videos on adults in established adulthood, aged 30 to 45, and found positive associations between engagement with cooking content, cooking confidence, an increased frequency of home cooking, and health-conscious dietary behaviors (Contribution 2).

According to a 2025 article by Zeng and colleagues, analyzing how authority cues and storytelling techniques shape the credibility of nutrition videos in Australia, a significant share of the social media videos reviewed contained inaccurate or misleading nutrition information, often promoting restrictive or unbalanced diets. The assessment is based on the quality and accuracy of nutrition-related content on the TikTok app, focusing on the popular “#WhatIEatinaDay” trend (Contribution 3).

According to the 2025 systematic review carried out by Athsnasoula et al., social media often promotes unhealthy eating behaviors among adolescents, such as the increased consumption of fast food and sugar-sweetened beverages, raising concerns about its impact on young people’s eating habits (18–30). The narrative review identified consistent associations between high social media use and increased body dissatisfaction, the internalization of thin or muscular ideals, dieting behaviors, and disordered eating symptoms. Visual-based platforms such as Instagram and TikTok were identified as especially impactful due to their emphasis on appearance-focused content (Contribution 4).

Finally, the systematic review by Gamito et al. (2025), based in Portugal, indicated that health care providers primarily use these platforms for nutrition education, health promotion, and countering misinformation. The review, which drew on studies from multiple countries, examined how nutritionists and dietitians use social media to communicate with the public. While professional engagement on social media was shown to strengthen nutritional literacy and public trust, the review also emphasized continuing challenges, including limited regulation, time constraints, and competition from non-credentialed influencers (Contribution 5).

## 4. Conclusions

Overall, this Special Issue indicates that social media exposure is associated with changes in food consumption patterns, which may be either healthy or unhealthy depending on who creates, curates, and distributes the content. Targeted, peer-proximate, and literacy-enhancing content embedded within well-designed interventions shows promise for improving diet quality. The included studies also reflect regional diversity and highlight current trends in social media use, with video-based platforms such as Instagram and TikTok being predominantly used. Therefore, future research should prioritize longitudinal and experimental study designs, incorporating age-stratified analyses, to better clarify developmental vulnerabilities and optimize intervention targeting.
